# Changes in the Th1 : Th2 Cytokine Bias in Pregnancy and the Effects of the Anti-Inflammatory Cyclopentenone Prostaglandin 15-Deoxy-Δ^12,14^-Prostaglandin J_2_

**DOI:** 10.1155/2012/416739

**Published:** 2012-05-29

**Authors:** Lynne Sykes, David A. MacIntyre, Xiao J. Yap, Sathana Ponnampalam, Tiong Ghee Teoh, Phillip R. Bennett

**Affiliations:** ^1^Parturition Research Group, Department of Surgery and Cancer, Institute of Reproduction and Developmental Biology, Imperial College London, W120NN London, UK; ^2^St Mary's Hospital, Imperial College Healthcare NHS Trust, W21NY London, UK

## Abstract

Pregnancy is a complex immunological state in which a bias towards T helper 2 (Th2) protects the fetus. Evidence suggests that proinflammatory cytokines increase the risk of poor neonatal outcome, independently of the direct effect of preterm labour. The anti-inflammatory prostaglandin 15-deoxy-Δ^12,14^-Prostaglandin J_2_ (15dPGJ_2_) inhibits nuclear factor Kappa B (NF-**κ**B) in amniocytes and myocytes *in vitro *and is a ligand for the chemoattractant receptor-homologous molecule expressed on Th2 cells (CRTH2) receptor. Here we examine the Th1:Th2 cytokine bias in pregnancy and whether 15dPGJ_2_ could be used to inhibit the production of the proinflammatory cytokines through inhibition of NF-**κ**B while simultaneously promoting Th2 interleukin 4 (IL-4) synthesis via CRTH2 in T helper cells. Peripheral blood mononuclear cells (PBMCs) from women at 28 weeks, term pre-labour, term labour as well as non-pregnant female controls were cultured with 15dPGJ_2_ or vehicle control and stimulated with phorbol myristyl acetate (PMA)/ionomycin. The percentage of CD4^+^ cells producing interferon gamma (IFN-**γ**) and tumor necrosis factor alpha (TNF-**α**) in response to PMA/ionomycin was significantly reduced in pregnancy. 15dPGJ_2_ reduced IFN-**γ** and TNF-**α** production in stimulated T helper cells, but did not alter IL-4 production in CRTH2^+ve^ cells. 15dPGJ_2_ also reduced phospho-p65 in stimulated PBMCs. In summary, 15dPGJ_2_ suppresses the Th1 response of PBMCs during pregnancy and active labour whilst maintaining the Th2 response suggesting a therapeutic benefit in reducing neonatal morbidity in inflammation-induced PTL.

## 1. Introduction

Preterm labour <34 weeks occurs in about 4% of pregnancies [1]. In 80–85% of cases of spontaneous preterm labour (PTL) <28 weeks, there is evidence of intrauterine infection [2]. Despite increased awareness of the association between inflammation and preterm labour [3], there have been no major advances in the prevention of preterm labour which have been shown to improve long-term neonatal outcomes. During normal term labour, the uterus and cervix become infiltrated with leukocytes and undergo changes in response to local secretion of proinflammatory cytokines. A similar pattern of biochemical events occur during PTL; however, the triggers for this premature activation are still not fully understood. Regardless, the presence of increased proinflammatory cytokines during pregnancy is clearly associated with a poor prognosis for babies born preterm [4].

The immune system can generally be divided into the innate and adaptive immune system. The former is a nonspecific system providing immediate defence against pathogens, while the latter is more targeted, characterised by T and B lymphocytes. Although cross-talk between these lymphocytes exist, B cells and their antibodies mainly give rise to humoral immunity, whereas T cells primarily provide cell mediated immunity [5]. T helper cells (CD4^+^) form a subset of T cells and can be further subdivided into T helper 1 cells (Th1) and T helper 2 cells (Th2) depending on their pattern of cytokine production. Th1 cells secrete pro inflammatory cytokines such as interferon gamma (IFN-*γ*) and tumor necrosis factor alpha (TNF-*α*), whereas the Th2 cells secrete anti-inflammatory cytokines such as interleukin 4 (IL-4), IL-10, and IL-13 [6]. A mutually exclusive interaction exists between the Th1 interleukin, IFN-*γ*, and the Th2 interleukin, IL-4. IL-4 is the dominant factor for promoting growth and differentiation from the Th0 to the Th2 subtype, and directly inhibits the development of the Th1 cells [7]. IFN-*γ* indirectly promotes Th1 differentiation by upregulating the IL-12 receptor whilst inhibiting the growth of Th2 cells [8, 9].

Wegmann and colleagues first developed the concept that during pregnancy there is a shift from a T helper 1 (Th1) response to a T helper 2 (Th2) bias during pregnancy that functionally induces maternal tolerance and suppression [10]. Consistent with this notion, administration of the Th1 interleukins IFN-*γ* [11] and IL-2 [12] leads to fetal loss and preterm labour in the mouse. Similarly, CBA × DBA/2 mice that have placentas deficient in IL-4 and IL-10 are prone to fetal resorption. Treatment of these mice via intraperitoneal injection of IL-10 protects the fetuses from resorption [13]. Several human studies have shown a Th2 bias in the ratio of circulating T helper cytokine profile in normal pregnancy; and an increase in the Th1 ratio in cases of recurrent miscarriage [14] and in preeclampsia [15]. Peripheral blood lymphocytes taken from women in the first trimester show an increased production of IL-4 and IL-10 and less IFN-*γ* compared to nonpregnant controls *in vitro *[16]. There is *in vivo *evidence in pregnant women of increased expression of IL-4 messenger ribonucleic acid (mRNA) and lower expression of the IFN-*γ* mRNA [17].

Not all studies support the requirement of the shift from Th1 to Th2 for successful pregnancy outcome [18, 19]. Despite showing a suppression of IFN-*γ* and an increase in IL-10 during pregnancy compared to nonpregnant controls, Bates et al. showed no difference in IFN-*γ*, IL-10, or IL-4 secretion in women who subsequently miscarried compared with those who went on to complete their pregnancy [18]. However, contrary to this study, women with recurrent miscarriage have been shown to have increased IFN-*γ* and TNF-*α* levels compared with women that go on to have successful pregnancies [20]. While the mechanism regulating the Th1 : Th2 ratio is yet to be fully elucidated the importance of maternal immune tolerance during pregnancy is unquestionable. Several pregnancy-related proteins are known to promote Th2 bias such as leukemia inhibitory factor [21], progesterone, progesterone-induced blocking factor [22], and estradiol [23]. Prostaglandin D_2_ (PGD_2_) promotes IL-4, IL-13, IL-5, and IL-10 production in T helper 2 cells *in vitro *via the second PGD_2_ receptor; chemoattractant receptor-homologous molecule expressed on Th2 cells (CRTH2) [24]. PGD_2_ is produced by the placenta and may play a role in the chemoattraction of Th2 cells via the CRTH2 receptor to the maternal fetal interface to produce a localised Th2 bias [25].

15-deoxy-Δ^12,14^-Prostaglandin J_2_ (15dPGJ_2_), an endogenous prostaglandin (PG), is a product of PGD_2_ via a series of dehydration reactions [26]. As well as being a ligand for Peroxisome proliferator-activated receptor gamma (PPAR*γ*), it is also an agonist of the CRTH2 receptor [27]. We have previously demonstrated that this anti-inflammatory PG inhibits nuclear factor Kappa B (NF-*κ*B) in amniocytes and myocytes *in vitro* [28] and delays infection induced preterm labour and increases pup survival in the mouse via NF-*κ*B inhibition [29]. Moreover, recent work has shown that the first trimester of pregnancy is associated with the suppression of available T cell NF-*κ*B and reduced levels of IFN-*γ* compared to nonpregnant controls [30, 31]. Collectively these data suggest that the Th1 : Th2 ratio is modulated through the regulatory interplay of both Th1 suppression and Th2 promotion.

In this study, we examined the expression of the dominant Th1 interleukins IFN-*γ* and TNF-*α*, and the Th2 interleukin IL-4 in T helper cells obtained from nonpregnant women, women in the early and late third trimester, and during labour. We examined the effect of the CRTH2 agonist, 15dPGJ_2_, on these interleukins and on NF-*κ*B activation at different gestational time points to determine its potential role in suppressing the Th1 response via NF-*κ*B whilst simultaneously promoting the Th2 response via CRTH2.

## 2. Materials and Methods

### 2.1. Subjects

Pregnant women at 28 weeks and term (prelabour and in labour) and nonpregnant women of child bearing age were included in the study in accordance with ethical approval from the South East London Ethical Committee Ref. 10/H0805/54. Exclusion criteria included women with Asthma and HIV, women not of childbearing age, and women with a fever. After obtaining informed consent, 5 mL of peripheral venous blood was collected in sodium citrate tubes, and processed within 30 mins of collection. Unless otherwise stated, a minimum of 4 subjects were included for each experimental sample group.

### 2.2. Isolation of Peripheral Blood Mononuclear Cells (PBMCs)

Blood was diluted 1 : 1 with phosphate-buffered saline (PBS) and carefully layered onto Ficoll-Paque PLUS (GE Healthcare, Uppsala, Sweden) before centrifuging at 400 ×g for 40 mins at room temperature. After centrifugation, the halo containing PBMCs was carefully transferred into a clean centrifuge tube and washed twice with 7 mL of PBS. After centrifugation (400 ×g for 10 mins), the cell pellet was resuspended in either PBS or RPMI 1640 (Invitrogen Life Technologies, Grand Island, NY, USA) culture medium.

### 2.3. CRTH2 Expression Studies

The PBMC pellet was resuspended PBS, and cells were counted with a Neubauer haemocytometer and then resuspended in staining buffer (1% fetal calf serum; 0.09% sodium azide in PBS) to obtain roughly 3.5 × 10^6^ cells per sample. Preparations were incubated in the dark for 1 h at 37°C with 20 *μ*L of CRTH2-phycoerythrin (CRTH2-PE) (Beckman Coulter, High Wycombe, UK) and 3 *μ*L of CD4-Allophycocyanin (CD4-APC) (BD Pharminogen, Oxford, UK) or the relevant isotype controls; Rat Immunoglobulin (Ig)G_2a_-PE (Beckman Coulter) and Mouse IgG_1*κ*_-APC (BD Pharminogen). After incubation, the PBMC suspension was washed twice in 1 mL of PBS and then resuspended in PBS for analysis. Flow cytometry settings were as follows: forward scatter *E*0 voltage, 1.00 Amp gain Lin, and side scatter of 329 voltage, 1.00 Amp gain Lin. A total of 50,000 cells were counted and gating was on lymphocytes based on the forward and side scatter.

### 2.4. Intracellular Cytokine Studies (IL-4, IFN-*γ*, and TNF-*α*)

For intracellular cytokine studies, cells were resuspended in RPMI 1640 (Invitrogen) media supplemented with 10% fetal calf serum, 2 mM/L L-glutamine, 100 U/mL penicillin, and 100 *μ*g/mL of streptomycin (Sigma, St. Louis, MO, USA) before being plated in 24-well plates and incubated for 18 h in 5% CO_2_/humidified air at 37°C. Following this, 10 *μ*g of brefeldin A (Sigma) was added to each well to immobilize the interleukins in the golgi apparatus. Cells were pretreated with 32 *μ*M of 15dPGJ_2_ (Cayman Chemicals, Ann Arbor, MI, USA) or dimethyl sulfoxide (DMSO) (Sigma) vehicle control for 2 h in the case of IFN-*γ* and TNF-*α* experiments or 1 h for IL-4. Following this 50 ng/mL of phorbol myristate acetate (PMA) and 0.5 *μ*g/mL of ionomycin were added for 4 h in the case of IFN-*γ* and TNF-*α* experiments or 5 h for IL-4. Prior to intracellular staining, cell surface staining with either CRTH2 and CD4, or CD4 antibodies alone, was performed as described in Section 2.3. Cells were then fixed with 2% paraformaldehyde (PFA) and incubated at 37°C for 15 mins in the dark. Cells were washed and resuspended in 0.5% saponin and incubated for 30 mins on ice in the dark to permeabilize the cells. After incubation, cells were washed and resuspended in 0.5% saponin for intracellular staining with the relevant antibody; IL-4 PE/Cy7 (BioLegend, San Diego, CA, USA), IFN-*γ*-(fluorescein isothiocyanate (FITC) or TNF-*α*-FITC (BD Biosciences, Franklin Lakes, NJ, USA). Appropriate isotype controls were used; PE/Cy7 Rat IgG_1*κ*_ (Biolegend), or FITC Mouse IgG_1*κ*_ (BD Biosciences, Oxford, UK). Cells were incubated in the dark for 1 h at room temperature for IL-4 staining or 20 mins on ice for IFN-*γ* and TNF-*α* staining. Finally, cell suspensions were washed with 0.5% saponin and resuspended in PBS for flow cytometry analysis as follows: Forward scatter *E*0 voltage; 1.00 Amp gain Lin; side scatter of 329 Voltage; 1.00 Amp gain Lin.

### 2.5. Flow Cytometry

Flow cytometry of lymphocytes was carried out using FACSCalibur flow cytometer (BD Biosciences) equipped with FACSCalibur software for analysis. The lymphocyte population was gated on the scatter plot as determined by the characteristic forward scatter (FS) and side scatter (SC) which indicates the cell size and shape. The analysis was of CD4^+^, CRTH2^+^, and CRTH2^+^/CD4^+^ in the CRTH2 expression studies and IL-4^+^, IL-4^+^/CRTH2^+^, TNF-*α* ^+^/CD4^+^, and IFN-*γ* ^+^/CD4^+^ for the intracellular cytokines. The expression levels of CRTH2, CD4, and intracellular cytokines were evaluated by the percentage of cells expressing the protein of interest or by mean fluorescence intensity (MFI).

### 2.6. Sodium Dodecyl Sulfate Polyacrylamide Gel Electrophoresis (SDS-PAGE) and Western Blotting

Following culture and treatments, cells were collected using a 1 mL pipette and incubated on ice in whole cell lysis buffer (Cell Signalling, Beverly, MA, USA) with 5 *μ*L/mL of protease inhibitor (Sigma) lysis buffer for 5 mins and centrifuged for 20 mins at 13,000 rpm at 4°C. The supernatant was stored at −80°C until use. Prior to SDS-PAGE, protein concentrations were determined using the BIORAD quantification assay measuring absorbance at 655 nm. Approximately 15 *μ*g of extracted protein per sample was resolved by SDS-PAGE and subsequently transferred onto polyvinyl difluoride (PVDF) membranes (GE Healthcare) 100 V constant at 4°C. Following transfer, the membrane was then blocked in 5% (wt/vol) milk in tris-buffered saline (TBS) supplemented with 0.1% Tween 20 (TBST) for 1 h. The membrane was then probed with phospho-p65 (Ser 536) (Cell signalling) primary antibody (1 : 1000 in tris-buffered saline) overnight at 4°C followed by the appropriate secondary antibody (1 : 2000 in 1% milk/TBS) for 1 h at room temperature. Chemiluminescence detection was then carried out with ECL Plus (GE Healthcare), and the membranes developed using a high-performance chemiluminescence film (GE Healthcare). Blots were scanned, and densitometry was performed with ImageJ (v1.44p).

### 2.7. Statistical Analysis

Experimental sample groups consisted of at least 4 biological replicates unless otherwise stated. Statistical analysis was performed with Graph-Pad Prism (v4.0; GraphPad Software, San Diego, CA, USA). Paired Student's *t*-test or repeated measures ANOVA was conducted where appropriate. Samples with *P* < 0.05 was considered to be statistically significant.

## 3. Results

### 3.1. Gestational Effect on Th1 and Th2 Cytokines

**Figure 1:**
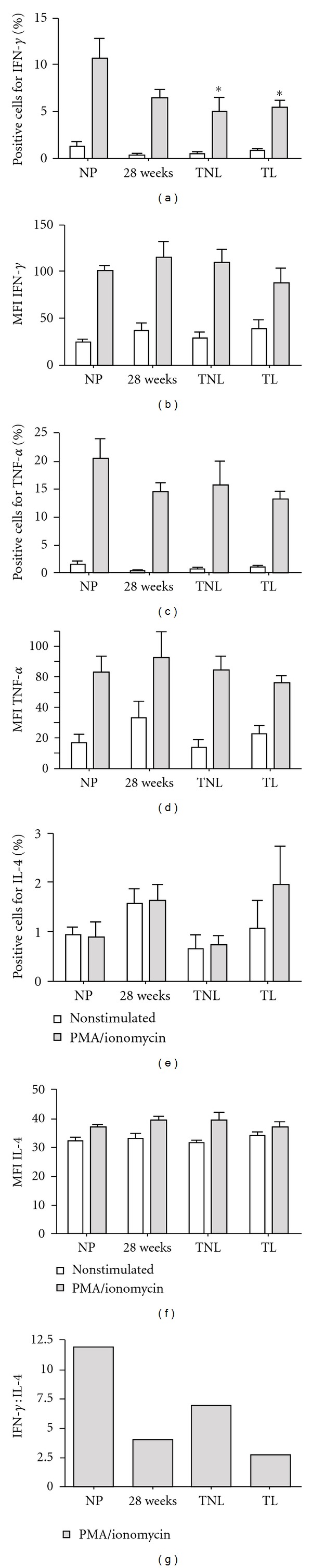
Th1 and Th2 cytokine production in peripheral T cells from nonpregnant and pregnant women. Peripheral blood mononuclear cells were isolated and stimulated with PMA/ionomycin. The percentage of CD4^+^/IFN-*γ* ^+^, CD4^+^/TNF-*α* ^+^, and CRTH2^+^/IL-4 positive cells were detected by flow cytometry in samples derived from nonpregnant (NP), 28-week pregnant, term no labour (TNL), and term labour (TL) samples (a), (c), and (e). A reduction in the percentage of peripheral CD4 positive cells secreting the Th1 cytokines IFN-*γ* and TNF-*α* was observed, whereas the percentage of cells secreting the Th2 cytokine, IL-4, remained consistent throughout gestation. Mean fluorescence intensity (MFI) was also assessed as a measure of total cytokine production (b), (d), and (f). PMA/ionomycin stimulation induced both Th1 and Th2 cytokine production in all sample groups compared to NL controls. No differences in gestation-dependent responses were detected. (g) a ratio of IFN-*γ* : IL-4 revealed a decrease in the Th1 : Th2 ratio during pregnancy, which was increased prior to the onset of labour. For statistical analysis ANOVA with Dunnett's multiple comparison test with NP as a control was used; **P < 0.01 and *P < 0.05.

A change in the production of the Th1 and Th2 cytokines in pregnancy has previously been described. In this study, we employed flow cytometry to examine the effect of stimulation by the mitogen PMA/ionomycin on cytokine production at different gestational stages of pregnancy and during labour (see Figure 1). Th1 cytokine profiles of CD4 positive cells were assessed for intracellular IFN-*γ* and TNF-*α* (Figures 1(a)–1(d)) and compared to levels of nonpregnant controls. The percentage of peripheral T cells producing IFN-*γ* in response to stimulation reduced in pregnancy from 10.7% in nonpregnant women to 6.7% at 28 weeks, 5.1% at term (*P* < 0.05), and 5.6% at term in labour (*P* < 0.05). Similarly, the proportion of TNF-*α* producing cells was reduced, although not significantly, from 20.6% in nonpregnant women to 14.5% at 28 weeks, 15.8% at term and 13.3% at term in labour. Overall levels of Th1 cytokine production (expressed as mean fluorescence intensity), in the CD4^+^/IFN-*γ* ^+^ or CD4^+^/TNF-*α* ^+^ cells remained consistent throughout gestation and unchanged compared to nonpregnant controls. The Th2 cytokine, IL-4, was similarly assessed in CRTH2 positive cells (Figures 1(e) and 1(f)). While PMA/ionomycin stimulation did not increase the percentage of IL-4 expressing cells, the mean fluorescence intensity of IL-4 was significantly increased in samples collected from women at 28 weeks (39.3, *P* < 0.01) and at term (39.4, *P* < 0.05) compared to levels of nonpregnant controls (37.1). Levels of IL-4 in term labouring samples were consistent with nonlabouring samples (37.1). The ratio of the IFN-*γ* : IL-4 producing cells reduces during pregnancy, due to the suppression of the Th1 rather than the promotion of the Th2 cytokine production (Figure 1(g)).

### 3.2. CRTH2 Expression

**Figure 2:**
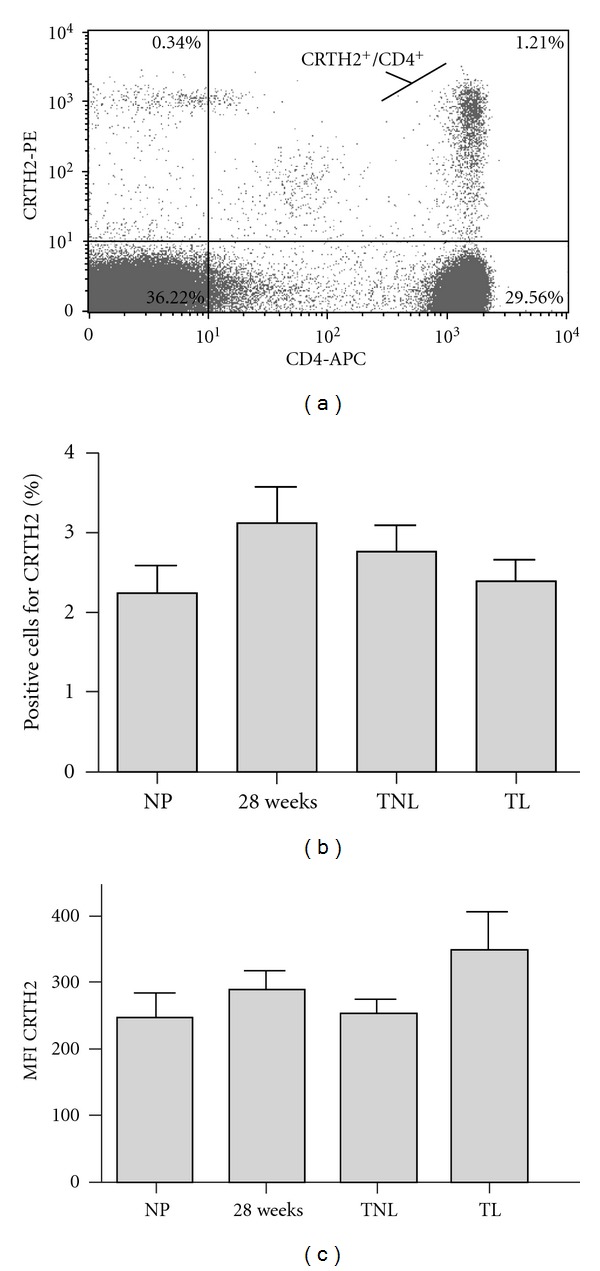
CRTH2 expression in lymphocytes derived from nonpregnant and pregnant subjects. Lymphocytes were gated according to forward scatter and side scatter and T helper cells were identified using CD4 as a cell surface marker (*n* = 8 for each group). A representative cytogram from a woman of 28-week gestation is presented with the right upper quadrant showing CRTH2^+^/CD4^+^ lymphocytes (a). The percentage of CRTH2 positive T helper cells (presented as a percentage of positive CD4 positive cells) increased slightly in the third trimester, although statistical significance was not reached (b). No change in mean fluorescence intensity was detected in either the nonpregnant or pregnant samples (c). For statistical analysis ANOVA with Dunnett's multiple comparison test with NP as a control was used.

To determine whether the Th2 response in pregnancy is reflected by an increase in the percentage of CRTH2 positive cells, we used CD4 as a marker of T helper cells and calculated the percentage these cells that express CRTH2 (Figure 2(a)). The percentage of CRTH2 positive cells in the CD4 positive population was observed to increase in the second trimester of pregnancy from 2.24% in nonpregnant women to 3.12% at 28 weeks before reducing to 2.75% at term and 2.4% in term labour, although statistical significance was not reached (Figure 2(b)). There was no significant increase in the mean fluorescence intensity of CRTH2 in T helper cells between nonpregnant and pregnant subjects (Figure 2(c)), although a slight increase in CRTH2 production was noted in term labouring samples (*P* = 0.15).

### 3.3. The Effect of 15dPGJ_2_ on the Th1 and Th2 Cytokines

**Figure 3:**
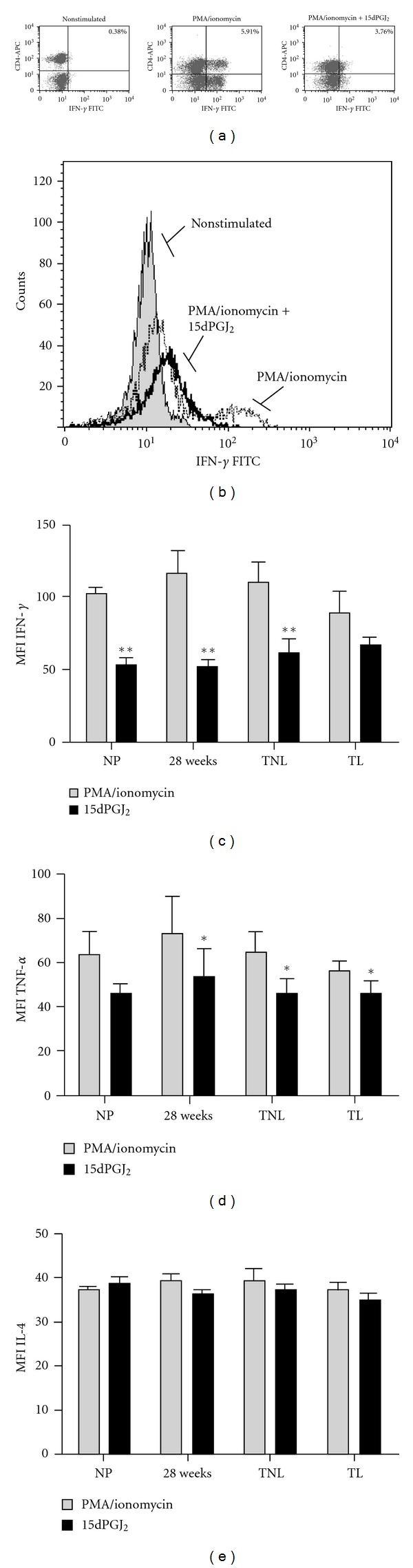
Effect of 15dPGJ2 on Th1 and Th2 cytokines. Peripheral blood mononuclear cells were incubated with vehicle control or 32 *μ*M of 15dPGJ_2_ and stimulated with PMA/ionomycin before being gated on the lymphocyte population according to forward and side scatter. A representative 28-week patient sample is presented with CD4^+^/IFN-*γ* ^+^ cells shown in the right upper quadrant (a). A representative histogram reveals a clear shift to the right upon stimulation indicative of increased IFN-*γ* producing cells (b). This effect was attenuated with 15dPGJ_2_ pre-treatment. PMA/ionomycin induced IFN-*γ* and TNF-*α* production was decreased with 15dPGJ_2_ treatment (c) and (d); however, levels of IL-4 remained unchanged. No change in IL-4 producing cells was detected (e). For statistical analysis a Student's *t*-test was used to compare means between paired treated and nontreated samples; **P < 0.01 and *P < 0.05.

15dPGJ_2_ is an anti-inflammatory PG and a ligand for CRTH2. We therefore explored the effect of 15dPGJ_2_ on the Th1 cytokines, IFN-*γ* and TNF-*α*. To do this, cells were preincubated with 32 *μ*M of 15dPGJ_2_ or vehicle control prior to the addition of PMA/ionomycin before intracellular cytokines were detected by flow cytometry (Figures 3(a) and 3(b)). 15dPGJ_2_ reduced the production of PMA/ionomycin stimulated IFN-*γ* production (measured as mean fluorescence intensity) in T helper cells from 101.9 to 53 (*P* < 0.01) in nonpregnant controls, 115.7 to 52.4 (*P* < 0.01) in 28 week samples and from 110 to 60.7 in term (*P* < 0.01) samples. There was a nonsignificant decrease observed in term labouring samples also (88.3 to 66.3, Figure 3(c)). Similarly, a significant decrease in PMA/ionomycin-induced TNF-*α* production following treatment with 15dPGJ_2_ was detected in all pregnant samples assessed (Figure 3(d)).

The percentage of T helper cells producing IFN-*γ* was also reduced from 10.7% to 5.13% in nonpregnant women (*P* < 0.01), 6.6% to 3.4% at 28 weeks (*P* < 0.01), 5.1% to 2.5% at term (*P* < 0.05), and 5.6% to 2.9% (*P* < 0.01). A reduction in the percentage of T cells producing TNF-*α* was also seen with 15dPGJ_2_ from 20.6% to 16.7% in nonpregnant women, 14.5% to 9.0% at 28 weeks (*P* < 0.05), 15.8% to 12.9% at term (*P* < 0.01), and 13.2% to 8.93% at term in labour (*P* = 0.05) (data not shown).

Considering its anti-inflammatory properties, it was hypothesized that 15dPGJ_2_ would induce an increase in IL-4 production via its action on the CRTH2 receptor. Contrary to what was anticipated, 15dPGJ_2_ had no effect on the production of IL-4 (Figure 3(e)). Consistent with this, the percentage of CRTH2+/IL-4+ lymphocytes remained constant following 15dPGJ_2_ treatment except for samples collected at 28 weeks gestation (1.65% to 1.16%, *P* < 0.05; data not shown).

### 3.4. 15dPGJ_2_ Inhibits PMA/Ionomycin-Stimulated NF-*κ*B Activation in Peripheral Blood Mononuclear Cells

**Figure 4:**
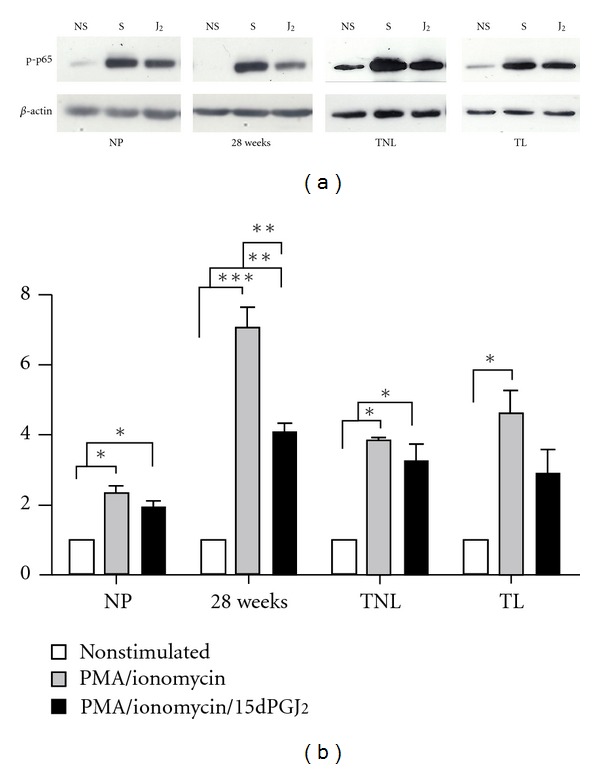
The effect of 15dPGJ2 on NF-*κ*B p65 phosphorylation in peripheral blood mononuclear cells. PBMCs were treated as previously described before being extracted and levels of phosphorylated p65 (p-p65) examined using immunoblotting. Representative immunoblots are shown for each gestational time point (a). Immunoblots were reprobed for *β*-actin as an internal loading control. Densitometric analysis of the immunoblots was conducted revealing a significant increase in p-p65 levels upon stimulation with PMA/ionomycin in all samples (b). A significant decrease in PMA/ionomycin stimulated p-p65 was observed following 15dPGJ_2_ treatment in 28-week samples. The capacity of 15dPGJ_2_ to inhibit PMA/ionomycin-induced p-p65 was lost in those samples collected at term. NS: nonstimulated, S: PMA/ionomycin stimulated, and J_2_: 15dPGJ_2_ pretreatment. Effect of treatment was examined for statistical significance at each gestational timepoint using ANOVA with Bonferroni's multiple comparison test; ***P < 0.001, **P < 0.01 and *P < 0.05.

Peripheral blood mononuclear cells were incubated with vehicle control or 32 *μ*M 15dPGJ_2_ and stimulated with PMA/ionomycin for 10 min before being extracted and assessed for levels of phosphorylated p65, the transcriptionally active subunit of NF-*κ*B. PMA/ionomycin treatment stimulated NF-*κ*B phosphorylation in all samples and at all gestational time points (Figure 4). Pretreatment of cells with 15dPGJ_2_ significantly reduced levels of p-p65 in samples collected from women at 28-week gestation. A clear trend of decreased p-p65 following 15dPGJ_2_ was observed in the remaining samples yet they did not return to basal levels of p-p65.

## 4. Discussion

The immunological paradox of pregnancy relies on a careful balance of both immune tolerance and immune suppression. Work by Medawar, and later Wegmann and colleagues, has led to the proposal that this balance is determined by a functional immune suppression via a shift from a Th1 response to a Th2 bias during pregnancy [10, 20, 32]. Although not all human and murine studies support the importance or the simplification of the Th1 : Th2 paradigm [18, 19, 33], further understanding of this hypothesis to aid the development of new therapeutic strategies is still required. In this study, we have explored the Th1 cytokines IFN-*γ* and TNF-*α*, and the Th2 cytokine IL-4 throughout pregnancy, and examined the potential of the cyclopentenone PG CRTH2 agonist, 15dPGJ_2_, to act as a therapeutic agent to modulate the Th1 : Th2 response.

Consistent with the notion of suppressed Th1 cytokine response throughout gestation [16, 17, 34, 35], our results revealed a reduction in the percentage of IFN-*γ* and TNF-*α*-secreting T helper cells during pregnancy in response to PMA/ionomycin stimulation (Figures 1(a) and 1(c)). However, mean production of IFN-*γ* and TNF-*α* remained constant within the CD4^+^/IFN-*γ* ^+^ and CD4^+^/TNF-*α* ^+^ cell populations (Figures 1(b) and 1(d)). This indicates that while fewer cells maintain the capacity to initiate a Th1 response to inflammatory stimuli with advancing gestation, a compensatory mechanism facilitates increased IFN-*γ* and TNF-*α* production. These results suggest that Th1 response during gestation is likely to be regulated at both an individual cellular level as well as at the level of the cell population.

As normal human labour reflects a proinflammatory state, we anticipated that the percentage of IFN-*γ* and TNF-*α* producing T helper cells would increase with the onset of labour. However, no difference between term nonlabouring and term labouring samples was detected. This may be due to the proinflammatory state of labour being a localized response in the uterus and fetal membranes, rather than an extending to peripheral inflammation. This would be consistent with the localised increase in the DNA binding activity of NF-*κ*B in amnion cells postlabour compared to prelabour [36] and in myometrium during labour compared to pre-labour [37] as well as the reported increase in labour of IFN-*γ* in amnion, choriodecidua, and placenta [38], and TNF-*α* in the amniotic fluid, myometrium, and cervix [39, 40] rather than in the peripheral blood.

A number of studies have shown that mitogen stimulation can induce IL-4 production in PBMCs of the second and third trimesters of pregnancy *in vitro* [16, 35, 41]. Intrinsic IL-4 production by PBMCs also shows an increase in pregnancy compared with nonpregnant controls [17, 42]. We therefore hypothesized that PMA/ionomycin stimulation of PMBCs would drive IL-4 production during pregnancy, and that this phenomenon would subside at term concurrent with the activation of inflammatory pathways involved in normal labour pathways. To examine any evidence of a shift towards the Th2 profile in peripheral blood during pregnancy, PBMCs were gated for CRTH2 (a marker for Th2 cells) and the level of IL-4 assessed (Figures 1(e) and 1(f)). The percentage of CRTH2^+^/IL-4^+^ secreting cells, as well as mean production levels of IL-4 remained consistent throughout pregnancy and advancing gestation (Figures 1(f) and 1(e)) although a slight increase in the percentage of IL-4 secreting cells was observed in both nonstimulated and stimulated 28-week samples. Between 0.4–6.5% of peripheral blood CD4^+^ cells of nonpregnant women express CRTH2 and secrete IL-4, but not IFN-*γ*, in response to PMA and ionomycin and are thus typical of Th2 cells [43]. Consistent with this, we found that between 2–4% of CD4^+^ PMBCs express CRTH2 (Figure 2(a)). This percentage was maintained throughout gestation with a minor, statistically nonsignificant, increase observed at 28 weeks (Figure 2(b)). Tsuda et al. have previously reported an increase in CRTH2 expression in CD4^+^ cells between nonpregnant (2.08%) and 7-week gestation (3.43%, *P* = 0.02) [44], but it is possible that this effect is diminished with advancing gestation. Mean levels of CRTH2 expressed in CD4^+^ cells were also unchanged through gestation and labour (Figure 2(c)). Taken together with the fact that we were unable to detect any difference in IL-4 secretion levels in these cells upon mitogen stimulation, our results collectively suggest that the Th2 response in PMBCs is maximised during pregnancy and cannot be further stimulated. This would imply that any beneficial effects of therapeutic agents intended to push the Th2 : Th1 ratio towards Th2 would need to do so through inhibition of Th1, rather than upregulation of Th2. Although the percentage of CRTH2 expressing CD4^+^ cells is low in the peripheral blood, we are unable to rule out any modest yet biologically significant changes in IL-4 production from these cells at a tissue-specific level.

To further investigate the potential of Th2 promotion versus Th1 suppression, we examined the response of PBMCs to 15dPGJ_2_, an endogenous product of Prostaglandin D_2_ (PGD_2_) (and potential therapeutic agent) that has been shown to promote Th2 bias through IL-4 production in T helper 2 cells *in vitro *via CRTH2 [24]. PGD_2_ is readily converted to 15dPGJ_2_ by a series of dehydration reactions in the presence of albumin and is found in the amniotic fluid [45], placenta [46], and urine [47]. 15dPGJ_2_ pretreatment significantly inhibited the PMA-stimulated production of both Th1 cytokines: IFN-*γ* and TNF-*α*, in nonpregnant and all pregnant samples (Figures 3(a)–3(d)). However, no effect on the production of the Th2 cytokine, IL-4, could be detected in response to 15dPGJ_2_ treatment. This may be due to an overriding effect of a non-CRTH2-mediated mechanism such as the inhibition of other signalling proteins involved the transcriptional regulation of IL-4 expression. Evidence for this has been previously reported in a PMA/Ionomycin-activated T-cell line, where 15dPGJ_2_ inhibited IL-4 production in a dose-dependent manner through the downregulation of nuclear factor of activated T-cells (NF-AT) activation [48].

The anti-inflammatory properties of 15dPGJ_2_ are thought to be at least partly via the inhibition of the transcription factor NF-*κ*B [49]. NF-*κ*B is known to play a key regulatory role in controlling the Th1 immune response by modulating the expression of Th1 cytokines [31]. NF-*κ*B activity in PBMCs during pregnancy is reduced compared to nonpregnant controls [50], and it has therefore been suggested that this is responsible for suppressed Th1 response in pregnancy [30]. To determine whether the observed inhibitory effects of 15dPGJ_2_ on Th1 cytokine production may be via NF-*κ*B, treated cells were extracted, and levels of the transcriptionally active NF-*κ*B subunit p-p65 were examined by immunoblotting. Our results indicate that 15dPGJ_2_ significantly inhibits p-p65 in PMA/ionomycin stimulated T helper at 28 weeks of gestation. A nonsignificant reduction was also observed in nonpregnant controls and samples acquired at term. This data provides evidence that the Th1 inhibitory properties of 15dPGJ_2_ are likely regulated through NF-*κ*B.

15dPGJ_2_ is a cyclopentenone PG which possesses an *α*,*β*-unsaturated carbonyl group in the cyclopentenone ring [51]. This group can form Michael adducts with I Kappa B kinase (IKK) [52], and the p65 [53] and p50 subunits leading to modification of these proteins, thus inhibition of NF-*κ*B activity [51]. We have shown that in amnion and myometrial cells, inhibition of NF-*κ*B by 15dPGJ_2_ is not via PPAR-*γ* [28]. However, 15dPGJ_2_-mediated suppression of Th1 interleukin expression in lymphocytes via PPAR-*γ* receptor inhibition or activator protein 1 (AP-1) has not been ruled out in this study.

**Figure 5:**
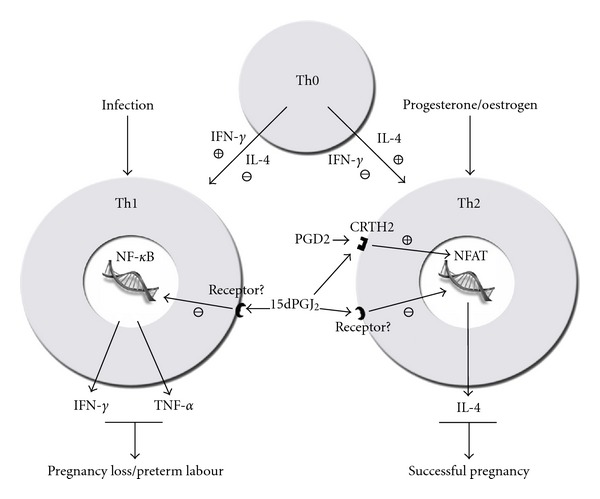
Schematic proposal of the Th1 and Th2 balance and the role of CRTH2 and NF-*κ*B. T-helper cell precursors are directed toward committed immunophenotype by the typical Th1 and Th2 interleukins, IFN-*γ* and IL-4, respectively. Infection or propregnancy hormones such as progesterone can further modulate the Th1/Th2 bias. Based upon our findings and those of others, we propose that 15dPGJ_2_ maintains a Th2 bias principally through the suppression Th1 interleukins through the inhibition of NF-*κ*B.

While 15dPGJ_2_ appears to have no effect on the peripheral Th2 response, the demonstrated inhibition of the Th1 response represents a collective shift toward a Th2 bias, or in other words, an increase in the Th2:Th1 ratio. This has important ramifications for the future design of therapeutic strategies to prevent preterm labour and pregnancy loss (Figure 5). The percentage of CRTH2 positive decidual lymphocytes is far higher, accounting for 10.5% of T lymphocytes and 18% of CD4+ lymphocytes [44]. Exploring the effect of 15dPGJ_2_ on purified decidual T cells may give rise both Th1 suppression and Th2 cytokine promotion.

Immunomodulating therapies such as progesterone (for women at high risk of preterm labour) and intravenous Immunoglobulins (for women with recurrent miscarriage) are currently being used in a clinical context. Progesterone suppresses IFN-*γ* production in peripheral blood mononuclear blood cells cultured with trophoblasts [54] and dydrogesterone suppresses both IFN-*γ* and TNF-*α* whilst increasing IL-4 in phytohaemagglutinin (PHA) stimulated PBMCs leading to a substantial Th1 to Th2 shift [55]. Immunoglobulins are capable of altering the Th1 : Th2 balance leading to a reduction in the CD4^+^IFN-*γ* ^+^/CD4^+^IL-4^+^ lymphocyte ratio [56]. The endogenous protein, progesterone-induced blocking factor (PIBF), is capable of increasing the production of IL-4 and IL-10 in PBMC, but has no effect on the Th1 cytokines IFN-*γ* and TNF-*α* [57].

15dPGJ_2_ may show more promising immunomodulating effects for the prevention of preterm labour exploiting its ability to both suppress the Th1 cytokines with the added benefit of inhibiting NF-*κ*B activity. Preterm labour, particularly in the presence of chorioamnionitis, is associated with a shift towards the Th1 cytokine response [58]. PBMCs of women in preterm labour when stimulated with PMA produce significantly higher IFN-*γ* compared to women who go on to deliver at term [59]. The presence of IFN-*γ* in cervicovaginal fluid in the late second and early third trimesters has also been shown to be a risk factor for preterm labour in asymptomatic women [60]. Similarly, TNF-*α* is thought to be key cytokine involved in the initial trigger of biochemical events leading to infection-mediated preterm birth, for example, by causing prostaglandin E_2_ (PGE_2_) synthesis by intrauterine tissues which drive pathways leading to uterine contractility and cervical ripening [61–63]. While global changes in the PBMCs likely reflect important systemic inflammatory events, their sub-components (e.g., T cells, B cells, and monocytes) may play functionally independent roles at the maternal fetal interface. Future work using an *in vivo* model examining effects of 15dPGJ_2_ and CRTH2 agonists on Th1 and Th2 interleukins at local sites (e.g., decidua and myometrium) would allow us to extrapolate how immune cells influence nonimmune cell function.

Prophylactic administration of 15dPGJ_2_ to women at high risk of preterm birth may lead to the inability of infection to activate NF-*κ*B and thus preventing transcription of labour associated genes, and the inability to alter the Th1 : Th2 bias to favour Th1, thus preventing the pro inflammatory detrimental environment of the fetus. Consideration for the route of administration should be supported by further studies of the potential local effect of 15dPGJ_2_ on interleukin production from the cervix, myometrium, and fetal membranes. The anti-inflammatory effect on NF-*κ*B and the Th1 interleukins may not be replicated in the cervix, for example, which would serve as the least invasive route of administration of 15dPGJ_2_ in the form of a pessary. Absorption at the local level is also unlikely to provide systemic levels high enough to effect peripheral cytokine production, likewise, the effect of peripheral administration on the local maternal fetal interface is not guaranteed to result in a physiological effect. Nevertheless, this study shows promising potential for the future application of 15dPGJ_2_ as an immunomodulating therapy for infection-mediated preterm labour, and thus further studies examining its effect on gestational tissues should be pursued.

## 5. Conclusion

This study presents evidence that the Th1 : Th2 balance is changed in pregnancy through Th1 suppression rather than Th2 promotion. We have also demonstrated the potential anti-inflammatory effects of 15dPGJ_2_ through the suppression of NF-*κ*B and the Th1 proinflammatory cytokines IFN-*γ* and TNF-*α*. These properties may be of therapeutic benefit for the prevention of infection-mediated preterm labour and the reduction of inflammation induced neonatal morbidity, where an aberrant profile favouring the Th1 response is seen. Thus, future work in this area may be best directed toward designing therapeutic strategies aimed to manipulate the Th1 response during pregnancy as a rational strategy for the prevention of preterm labour and pregnancy loss/miscarriage.
